# Effect of excluding fractured or abnormal vertebrae on the trabecular bone score measurement

**DOI:** 10.1007/s11657-024-01485-1

**Published:** 2024-12-27

**Authors:** Yen-Huai Lin, Michael Mu Huo Teng

**Affiliations:** 1 Department of Medical Imaging, Cheng Hsin General Hospital, 45 Cheng Hsin Street, Taipei, 112 Taiwan; 2 Department of Medicine, School of Medicine, National Yang Ming Chiao Tung University, Taipei, Taiwan

**Keywords:** Compression fracture, Degenerative abnormality, Systematic intervertebral variation, Trabecular bone score

## Abstract

***Summary*:**

Brief rationale: The use of L1–L4 vertebrae, without exclusions, has been recommended for trabecular bone score (TBS) measurements.

Main result: Excluding abnormal and fractured vertebrae affected the TBS.

Significance of the paper: Fracture or degenerative abnormality may not affect TBS. The preferred action may involve including all levels without exclusions.

**Purpose:**

The use of L1–L4 vertebrae, without exclusions, has been recommended for trabecular bone score (TBS) measurements. We aimed to investigate the effect of excluding fractured or abnormal vertebrae from TBS.

**Methods:**

Dual-energy X-ray absorptiometry images of 2767 participants, including 1080 without excluded vertebrae, 556 with fractured vertebrae, and 1131 with abnormal vertebrae showing a 1.0 T-score difference compared to the adjacent vertebrae, were retrospectively reviewed. Differences between TBS measurements with and without fractured or abnormal vertebrae were evaluated.

**Results:**

Among 1080 participants without excluded vertebrae, TBS was 1.234 at L1, 1.296 at L2, 1.308 at L3, and 1.301 at L4. A significantly higher mean TBS was seen after excluding L1, whereas a significantly lower mean TBS was seen after excluding L2–L4. In the 556 participants with fractured vertebrae, excluding the involved level from the TBS calculation led to a significant difference in the total sample, women, ≥ 70 years old, and overweight subgroups. A significantly higher mean TBS was seen after excluding the fractured L1, whereas a significantly lower mean TBS was seen after excluding fractures at L2–L4. Among the 1131 participants with abnormal vertebrae, excluding the involved level from the TBS led to a significant difference in age, sex, and body mass index subgroups. Excluding abnormal L1 and L4 vertebrae led to a significantly higher and lower mean TBS, respectively.

**Conclusion:**

Excluding fractured or abnormal vertebrae led to differences in TBS across various subgroups. Regarding the effect of vertebral level exclusion, the observed differences may be attributed to the systematic intervertebral variation, which is unrelated to any effect from fractures or degenerative abnormalities.

**Supplementary information:**

The online version contains supplementary material available at 10.1007/s11657-024-01485-1.

## Introduction

The National Institutes of Health defines bone strength as the combination of bone density and quality [1]. The International Society for Clinical Densitometry (ISCD) recommended the use of the trabecular bone score (TBS) to evaluate bone quality [2]. There is a natural trend in TBS measurements from L1 to L4, characterized by a lower TBS at L1 than at L2–L4 [3, 4]. TBS can be used in association with the Fracture Risk Assessment Tool (FRAX) and bone mineral density (BMD) to adjust for fracture risk. Thus, precise TBS measurements are important in routine clinical practice.

According to the 2019 ISCD Adult Official Positions, anatomically abnormal vertebrae should be excluded from the analysis when measuring the spinal BMD using dual-energy X-ray absorptiometry (DXA) [5]. Two situations may lead to excluding vertebrae. First, vertebrae that are clearly abnormal and non-assessable, such as those with obvious compression fractures, should be excluded. Second, exclusion is warranted when there is a T-score difference more than 1.0 between the vertebra in question and the adjacent vertebrae is present, most commonly occurring in degenerative changes. Therefore, according to the 2019 ISCD criteria, fractured or abnormal vertebrae should be excluded from the analysis of spinal BMD. Similarly, fractured or abnormal vertebrae were also excluded from TBS measurements [6].

However, the 2023 updated ISCD Adult Official Positions recommended that the L1–L4 vertebrae should be used for TBS measurements without exclusions. These levels were also recommended for calculating TBS-adjusted FRAX probabilities, even in the presence of chronic lumbar compression fractures and moderate degenerative changes [7]. Thus, although fractured or abnormal vertebrae were excluded from TBS measurements in the past, it may be necessary to include them at present. The Osteolaus cohort reported that the TBS was not affected by vertebral exclusions [8], whereas the Manitoba BMD registry showed contrasting results [9]. Moreover, these studies did not demonstrate the separate effect of excluding fractured and abnormal vertebrae, which may affect TBS differently, from TBS measurements [8, 9]. Therefore, in this study, we aimed to investigate the separate effect of excluding fractured and abnormal vertebrae with a T-score difference of 1.0 compared to the adjacent vertebrae from TBS measurements.

## Methods

### Participants

DXA images of Chinese patients obtained at our hospital between 2019 and 2021 were retrospectively reviewed. In this study, we included men aged ≥ 50 years and postmenopausal women for whom vertebrae were excluded from the BMD analysis because of radiographically confirmed compression fractures at any site from L1 to L4 or abnormal vertebrae with a 1.0 T-score difference compared to the adjacent vertebrae. Patients who had undergone spinal hardware placement, laminectomy, or vertebroplasty at any site from L1 to L4 and had a body mass index (BMI) outside the 15–37 kg/m^2^ range were excluded. A total of 1687 participants with vertebral exclusion from BMD analysis were enrolled in this study. Those with radiographically confirmed spinal fractures were included in the fractured vertebrae group, and those without radiographically confirmed spinal fractures were included in the abnormal vertebrae group. For comparison, patients without excluded vertebrae within 1 year were also reviewed; finally, 2767 participants were enrolled in this study. A flow chart showing the case selection procedure is shown in Online Resources 1. The requirement of informed consent was waived owing to the retrospective nature of this study. This study was approved by the Institutional Review Board, and all methods were performed following relevant guidelines and regulations.

### Group allocation

The participants in our study were classified into three groups: the normal vertebrae group, without excluded vertebrae (1080 participants); the fractured vertebrae group (556 participants); and the abnormal vertebrae group, with a T-score difference of 1.0 compared to the adjacent vertebrae (1131 participants). To investigate the effect of excluding fractured vertebrae on the TBS measurement, other levels of abnormal vertebrae with a T-score difference of 1.0 compared to the adjacent vertebrae were still used for TBS measurement in the fractured vertebrae group. If the participants had more than one fractured vertebra, the most severely fractured vertebra was investigated and other fractured vertebrae were still used for TBS measurement. In contrast, the abnormal vertebrae group did not include participants in the fractured vertebrae group.

### Trabecular bone score

TBS was measured using the iNsight software (version 3.0.2.0; Medimaps, Geneva, Switzerland), which utilized the anteroposterior spine DXA images obtained using a DXA scanner (Horizon W; Hologic Inc., Bedford, MA). The TBS coefficient of variation was 2.00%.

### Body mass index measurements

The participants were instructed to wear light clothing when their weight and height were measured, which were subsequently used to calculate their BMI. According to the classification provided by the Health Promotion Administration of the Taiwanese government, study participants were categorized as follows: underweight (< 18.5 kg/m^2^), normal weight (≥ 18.5 kg/m^2^ to < 24 kg/m^2^), overweight (≥ 24 kg/m^2^ to < 27 kg/m^2^), and obesity (≥ 27 kg/m^2^) [10].

### Assessment of compression fractures

In this study, vertebral compression fractures were confirmed using radiological images. Although X-ray imaging is not specific enough to precisely grade compression fractures, it is the most easily available tool for assessing compression fractures compared to computed tomography or magnetic resonance imaging. The latest X-ray before DXA was retrospectively reviewed to grade compression fractures. The height reduction was measured by a single well-trained technologist to avoid interobserver variability in our study. These fractures were categorized into mild (< 25% reduction in height), moderate (25–40% reduction in height), and severe (> 40% reduction in height) grades using the semiquantitative method [11].

### Statistical analysis

The differences in TBS between measurements with and without fractured or abnormal vertebrae were evaluated using a paired *t*-test. All reported *p*-values were two-sided, and *p*-values < 0.05 were considered statistically significant. Statistical analysis was performed using IBM SPSS Statistics for Windows version 19.0 (IBM Corp., Armonk, NY).

## Results

This study included a total of 2767 participants, which were classified into three groups, and the mean TBS at each level is shown in Table 1. There was a lower mean TBS at L1 than at L2, L3, or L4 in the three groups, which is consistent with the systematic intervertebral variation in TBS measurements. In the fractured vertebrae group, 556 patients (90 men and 466 women) had compression fractures (Table 2). Among these patients, 71.2% were over 70 years old, 45.9% had normal weight, 51.3% had the L1 fracture, and 62.6% had the severe compression fracture. Table 2 also showed the differences in TBS measurements with and without the inclusion of vertebrae with compression fractures. After excluding the fractured vertebra, the TBS showed a significant difference in the total sample as well as in the women, participants ≥ 70 years old, and overweight subgroups. Regarding the fracture level, a significantly higher mean TBS was observed at L1, whereas at L2, L3, or L4, there was a significantly lower mean TBS after excluding the fractured vertebra from TBS measurements. The normal vertebrae group was analyzed to investigate the effect of excluding each normal vertebral level on the TBS measurement. Excluding a normal vertebral level from the TBS measurement could have the same effect as exclusions based on fractures (Online Resources 2). The TBS showed a significant difference in mild and severe fractures, but no significant change was observed in moderate fractures.

**Table 1:** Mean trabecular bone score at each level in the three groups

	Normal vertebrae group (*n* = 1080)		Fractured vertebrae group (*n* = 556)		Abnormal vertebrae group (*n* = 1131)	
	Mean	Standard deviation	Mean	Standard deviation	Mean	Standard deviation
L1	1.234	0.11	1.202	0.12	1.228	0.12
L2	1.296	0.11	1.249	0.11	1.280	0.11
L3	1.308	0.10	1.277	0.11	1.300	0.10
L4	1.301	0.10	1.267	0.10	1.305	0.10
L1–L4	1.285	0.09	1.248	0.08	1.278	0.09

**Table 2:** Comparison of the mean trabecular bone score including and excluding fractured vertebrae

			Including fractured vertebrae		Excluding fractured vertebrae		
	*n*	%	Mean	Standard deviation	Mean	Standard deviation	*p*
Total	556		1.248	0.08	1.244	0.09	0.001
Sex							
Men	90	16.2	1.297	0.08	1.295	0.09	0.441
Women	466	83.8	1.239	0.08	1.234	0.08	0.001
Age							
< 59	32	5.8	1.289	0.08	1.286	0.09	0.437
60–69	128	23.0	1.247	0.08	1.243	0.09	0.137
70–79	219	39.4	1.245	0.09	1.241	0.09	0.041
≥ 80	177	31.8	1.246	0.08	1.242	0.08	0.031
Body mass index							
Underweight	37	6.7	1.255	0.08	1.247	0.08	0.138
Normal	255	45.9	1.256	0.08	1.254	0.09	0.202
Overweight	150	27.0	1.249	0.08	1.241	0.08	0.003
Obesity	114	20.5	1.231	0.09	1.228	0.09	0.297
Fractured vertebra							
L1 fracture	285	51.3	1.245	0.08	1.253	0.08	< 0.001
L2 fracture	124	22.3	1.259	0.09	1.248	0.09	< 0.001
L3 fracture	95	17.1	1.242	0.07	1.215	0.08	< 0.001
L4 fracture	52	9.4	1.263	0.08	1.245	0.09	< 0.001
Fracture grading							
Mild fracture	38	6.8	1.260	0.08	1.251	0.09	0.018
Moderate fracture	170	30.6	1.251	0.08	1.249	0.09	0.207
Severe fracture	348	62.6	1.246	0.08	1.241	0.09	0.006

A total of 1131 participants had abnormal vertebrae with a T-score difference of 1.0 compared to the adjacent vertebrae. Among these participants, 63.4% were 65 years or older and 46.2% had normal weight. In addition, 12.8%, 4.5%, 3.4%, and 35.5% had abnormal L1, L2, L3, and L4 vertebrae, respectively. Table 3 shows the differences between TBS measurements with and without the inclusion of abnormal vertebrae. After excluding abnormal vertebrae, the TBS showed a significant difference in the total sample as well as in the men, women, participants ≥ 65 years old, normal weight, overweight, and obesity subgroups. With regard to the level of abnormal vertebrae, a significantly higher mean TBS was observed at L1, whereas a significantly lower mean TBS was noted at L4 after excluding abnormal vertebrae in the TBS measurement. Similar to the findings at L1, a significantly higher mean TBS was also found at the L1–L2 vertebrae. Conversely, a significantly lower mean TBS was observed at the L2–L3 and L3–L4 vertebrae. Excluding a normal vertebral level from the TBS measurement could have the same effect as the exclusion based on degenerative abnormalities (Online Resources 2).

**Table 3:** Comparison of the mean trabecular bone score including and excluding abnormal vertebrae

			Including abnormal vertebrae		Excluding abnormal vertebrae		
	*n*	%	Mean	Standard deviation	Mean	Standard deviation	*p*
Total	1131		1.278	0.09	1.270	0.09	< 0.001
Sex							
Men	167	14.8	1.335	0.08	1.328	0.09	0.007
Women	964	85.2	1.269	0.08	1.260	0.09	< 0.001
Age							
< 54	82	7.3	1.363	0.08	1.360	0.09	0.279
55–59	126	11.1	1.306	0.07	1.302	0.08	0.215
60–64	207	18.3	1.296	0.08	1.291	0.08	0.063
65–69	253	22.4	1.275	0.08	1.268	0.09	0.003
70–74	205	18.1	1.252	0.09	1.240	0.09	< 0.001
75–79	116	10.3	1.250	0.08	1.236	0.09	< 0.001
≥ 80	142	12.6	1.248	0.08	1.236	0.09	< 0.001
Body mass index							
Underweight	38	3.4	1.287	0.08	1.289	0.08	0.752
Normal	523	46.2	1.288	0.09	1.281	0.09	< 0.001
Overweight	319	28.2	1.279	0.09	1.270	0.09	< 0.001
Obesity	251	22.2	1.256	0.09	1.244	0.09	< 0.001
Abnormal vertebrae							
L1	145	12.8	1.256	0.09	1.276	0.09	< 0.001
L2	51	4.5	1.273	0.09	1.271	0.09	0.641
L3	39	3.4	1.293	0.08	1.287	0.09	0.083
L4	402	35.5	1.276	0.08	1.263	0.09	< 0.001
L1, L2	118	10.4	1.286	0.10	1.296	0.10	0.005
L1, L3	8	0.7	1.252	0.09	1.235	0.12	0.539
L1, L4	75	6.6	1.254	0.09	1.264	0.10	0.065
L2, L3	43	3.8	1.321	0.10	1.291	0.10	< 0.001
L2, L4	42	3.8	1.295	0.10	1.294	0.10	0.902
L3, L4	208	18.4	1.290	0.08	1.256	0.10	< 0.001

## Discussion

In the past, anatomically abnormal vertebrae were excluded from the analysis of spinal BMD and TBS. However, present recommendations state that all vertebrae should be included in TBS measurements. After excluding fractured or abnormal vertebrae from TBS measurements, we found a significant difference within the age, sex, BMI, and vertebral level subgroups. Regarding the vertebral levels, there was a significant increase when L1 was excluded and a significant decrease when L4 was excluded, regardless of whether this was due to fracture or degenerative abnormality, indicating a general effect of the intervertebral variation in TBS.

In the Osteolaus cohort, there were 800 excluded vertebrae in 572 women, out of which 40 were due to grade 2 or 3 fractures [8]. The study combined fractured and abnormal vertebrae and reported that TBS remained the same in all age groups regardless of the exclusion of these vertebrae [8]. The Manitoba BMD registry also combined all vertebral exclusions and showed that TBS was affected by vertebral exclusions [9]. However, in this study, we investigated the separate effect of excluding abnormal or fractured vertebrae on TBS measurements in the 556 participants with fractured vertebrae and in the 1131 participants with abnormal vertebrae. Our study showed a significant difference within the age subgroups in both the fractured and abnormal vertebrae groups. The differences between our study and the Osteolaus cohort may be attributed to the inclusion of a larger number of participants in our study.

In 2023, the ISCD recommended the inclusion of fractured vertebrae in the TBS measurement. Our study showed that L1 was the most commonly fractured vertebra and that there was a significant difference among fractured vertebral levels. After excluding fractured vertebrae from the TBS measurement, a significantly higher mean TBS at L1 and a significantly lower mean TBS at L2, L3, and L4 were observed. Hsu et al. reported no significant difference in the mean TBS at L1 between the control and the fractured groups, even among different healing subgroups [12]. Additionally, there was only a minimal effect of lumbar fractures on the TBS measured from L1 to L4 in the Manitoba BMD Registry study [13]. Therefore, the observed differences may be attributed to the systematic intervertebral variation in TBS measurements, characterized by a lower TBS at L1 than at L2, L3, or L4 [3, 4]. Given that TBS measurements systematically increase from L1 to L4, it necessarily followed that excluding vertebral levels would lead to differences in the TBS measurement. Specifically, excluding fractured L1 would systematically increase the mean TBS value derived from the remaining vertebrae, whereas excluding fractured L4 would systematically decrease TBS derived from the remaining vertebrae. Excluding a normal vertebral level from the TBS measurement could have the same effect as excluding vertebrae based on the presence of fractures. Therefore, our findings may indirectly indicate that fractured vertebrae may not affect TBS measurements.

Regarding the group of abnormal vertebrae with a T-score difference of 1.0 compared to the adjacent vertebrae, L4 was the most commonly excluded vertebra, which was consistent with the findings of the Osteolaus cohort [8]. Dufour et al. and Kolta et al. both reported that TBS is unaffected by lumbar degenerative disease [4, 14]. A significantly higher mean TBS at L1 and a significantly lower mean TBS at L4 were found after excluding abnormal vertebrae from the TBS measurement, which was consistent with the fractured vertebrae group in our study. This is a general effect of the systematic intervertebral variation in TBS [3, 4]. Leslie et al. also reported the same results, which reinforced the findings of our study [9]. Excluding normal vertebrae from the TBS calculation could have the same effect as excluding vertebrae based on degenerative abnormalities, implying that degenerative abnormality may not play a role in TBS. Therefore, fracture or degenerative abnormality may not affect TBS, and the preferred action may be to include all levels.

TBS can be used in association with FRAX to adjust for fracture risk, and the adjustment is based on the TBS derived from L1 to L4 [15]. Excluding vertebral levels reflected a general effect of the intervertebral variation in TBS, altering FRAX-based or TBS-based risk classification [9, 16]. Leslie et al. developed fixed offsets to obtain accurate TBS-adjusted FRAX measurements for individuals with excluded vertebrae [9] and compared TBS-adjusted FRAX risk stratification derived from different combinations of vertebral levels [17]. However, this method is not currently implemented in the TBS software or clinical practice. Alternatively, including all levels without exclusion based on TBS calculated using the L1–L4 levels may be preferred since fractures or degenerative abnormalities may not affect the TBS.

A substantial number of studies have investigated TBS in women [8, 18]; however, there are limited data for men. In the group with abnormal vertebrae, we found a significant difference in TBS in both men and women subgroups. In addition to the sex subgroups, TBS was significantly lower in the normal weight, overweight, and obesity subgroups after abnormal vertebrae were excluded in the TBS measurement. However, in the group with fractured vertebrae, a significant difference was found only in overweight participants. Further studies are necessary to elucidate these differences. In our study, significant differences were observed within sex, age, BMI, and vertebral level subgroups depending on the exclusion of fractured or abnormal vertebrae.

The strength of this study lies in its inclusion of a substantial number of participants with fractured or abnormal vertebrae. We investigated the separate effect of excluding fractured and abnormal vertebrae on TBS measurements. Nevertheless, this study has some limitations. First, the participants were from an Asian population, potentially affecting the generalizability of our results. Second, the onset of compression fractures was unknown, and recent or old compression fractures could not be differentiated. Further studies are needed to compare the effect of the time since the compression fracture on TBS measurements. Third, not all individuals who underwent DXA also underwent X-ray imaging, and the abnormal vertebrae group might include those with unconfirmed fractures. However, in our country, pharmacological treatment at public expense would be prescribed when patients have radiographically confirmed fractures with DXA reports. Most individuals with spinal fractures had undergone X-ray. Therefore, there were few unconfirmed fractures in the abnormal vertebrae group.

## Conclusion

The 2023 ISCD recommendations stated that the L1–L4 vertebral levels should be used for TBS measurement without exclusions. Our study showed a significant difference in TBS within vertebral level subgroups after excluding fractured or abnormal vertebrae in the TBS measurements. A significantly higher mean TBS was found when L1 was excluded, but a lower mean TBS was obtained when L4 was excluded, regardless of whether this was due to fractures or degenerative abnormalities. This may be attributed to systematic intervertebral variation, and the preferred action may be to include all levels without exclusions. Further studies are needed to investigate whether including vertebrae with fractures or degenerative abnormalities in TBS measurements could enhance the performance of FRAX in predicting incident fractures.

## Supplementary information

Below is the link to the electronic supplementary material.

ESM 1 (DOCX 27.1 KB)

## Data Availability

The datasets used and analyzed during the current study are available from the corresponding author upon reasonable request.
