# Supplementary material for: Effect of excluding fractured or abnormal vertebrae on the trabecular bone score measurement
Source: Arch Osteoporos. 2024 Dec 27;20(1):4. doi: 10.1007/s11657-024-01485-1 (PMC11671424; doi:10.1007/s11657-024-01485-1)
Supplement: Supplementary file 1 — (DOCX 27.1 KB) [file 11657_2024_1485_MOESM1_ESM.docx]

**Online Resources 1. A flow chart of case selection**

Excluding those without excluded vertebrae on DXA (n=3,051)

DXA images between 2019 and 2021 (n=5,023)

Participants with spinal exclusion from the BMD analysis and whose X-ray were reviewed (n=1,687)

Fractured vertebrae group with radiographically confirmed spinal fractures (n=556)

Abnormal vertebrae group without radiographically confirmed spinal fractures (n=1,131)

Excluding those with spinal hardware, laminectomy, vertebroplasty, or BMI outside the 15–37 kg/m^2^ range (n=285)

Normal vertebrae group without excluded vertebrae within one year (n=1,080)

| **Online Resources 2. Trabecular bone score including and excluding each normal vertebra (n= 1,080)** | | | | | | |
| --- | --- | --- | --- | --- | --- | --- |
|  | Including all vertebrae | |  | Excluding each normal vertebra | |  |
|  | Mean | Standard deviation |  | Mean | Standard deviation | *p* |
| L1234 vs. L234 (excluding L1) | 1.285 | 0.09 |  | 1.301 | 0.09 | <0.001 |
| L1234 vs. L134 (excluding L2) | 1.285 | 0.09 |  | 1.281 | 0.09 | <0.001 |
| L1234 vs. L124 (excluding L3) | 1.285 | 0.09 |  | 1.277 | 0.09 | <0.001 |
| L1234 vs. L123 (excluding L4) | 1.285 | 0.09 |  | 1.279 | 0.09 | <0.001 |
